# Gas Dissolution Foaming as a Novel Approach for the Production of Lightweight Biocomposites of PHB/Natural Fibre Fabrics

**DOI:** 10.3390/polym10030249

**Published:** 2018-02-28

**Authors:** Heura Ventura, Luigi Sorrentino, Ester Laguna-Gutierrez, Miguel Angel Rodriguez-Perez, Monica Ardanuy

**Affiliations:** 1 Departament de Ciència dels Materials i Enginyeria Metal lúrgica (CMEM), Universitat Politècnica de Catalunya (UPC): C/Colom 11, TR4, 08222 Terrassa, Spain; monica.ardanuy@upc.edu; 2 Istituto per i Polimeri, Compositi e Biomateriali (IPCB), Consiglio Nazionale delle Ricerche (CNR): P/Enrico Fermi 1, Loc. Granatello, 80055 Portici, Italy; luigi.sorrentino@cnr.it; 3 Cellular Materials Laboratory (CellMat), Condensed Matter Physics Department, Facultad de Ciencias, Universidad de Valladolid (UVa): P° de Belén 7, 47011 Valladolid, Spain; ester.laguna@fmc.uva.es (E.L.-G.); marrod@fmc.uva.es (M.A.R.-P.)

**Keywords:** biopolymer, biocomposite, fabric reinforcement, natural fibres, foaming

## Abstract

The aim of this study is to propose and explore a novel approach for the production of cellular lightweight natural fibre, nonwoven, fabric-reinforced biocomposites by means of gas dissolution foaming from composite precursors of polyhydroxybutyrate-based matrix and flax fabric reinforcement. The main challenge is the development of a regular cellular structure in the polymeric matrix to reach a weight reduction while keeping a good fibre-matrix stress transfer and adhesion. The viability of the process is evaluated through the analysis of the cellular structure and morphology of the composites. The effect of matrix modification, nonwoven treatment, expansion temperature, and expansion pressure on the density and cellular structure of the cellular composites is evaluated. It was found that the nonwoven fabric plays a key role in the formation of a uniform cellular morphology, although limiting the maximum expansion ratio of the composites. Cellular composites with a significant reduction of weight (relative densities in the range 0.4–0.5) were successfully obtained.

## 1. Introduction

Over the last three decades, concern about the depletion of fossil resources, waste accumulation, and other environmental problems has prompted researchers to investigate biocomposite materials (of bio-based polymeric matrix and natural fibre reinforcement, for instance), which can offer sustainable and biodegradable replacements for conventional materials. In general, composites are appropriate for applications requiring light weight and good mechanical performance. Therefore, the development of cellular composites presents an interesting topic, since the combination of composite reinforcement with a cellular structure in the matrix can lead to a class of materials with high specific mechanical properties [1]. However, to our knowledge, the production of cellular biocomposites, with the combination of a biopolymer and a fabric reinforcement of natural fibres, has not been yet reported in the literature.

Among other biopolymers, polyhydroxyalkanoates (PHAs) offer a bio-based and biodegradable alternative. PHAs are bio-polyesters generated by bacteria under specific feeding conditions, and can be produced from sources that do not compete with food crops [2]. Although their properties are of great interest, their high price nowadays limits their competitiveness against the synthetic commodity polymers. Nonetheless, their use as matrix in cellular biocomposites presents an opportunity to increase their competitiveness, owing to a partial substitution of the matrix by natural fibres and gas, which reduces the amount of polymer required, and hence the cost. 

Reinforcements based on natural fibres (such as cellulosic fibres) have been shown to increase the stiffness and strength of PHAs [3,4,5], but a good fibre-matrix interaction (chemical, mechanical, or both) is required. Natural fibre reinforcements are often randomly dispersed short fibres, although they can also be used in the form of nonwoven fabrics [6,7]. This use of fibre mats instead of random fibres leads to a good distribution of fibres throughout the final structure while keeping a low production cost and allowing easy handling. Moreover, the mechanical reinforcement capacity of these fabrics allows the development of composites for structural applications.

To achieve a cellular structure in the composite’s matrix, a foaming process is required, where a blowing agent (such as carbon dioxide or nitrogen) is used to generate porosity, resulting in a lightweight cellular material. Nonetheless, the foaming process presents some challenges in this case. On one hand, PHAs present an intrinsic difficulty to be foamed owing to their low molecular weight, low melt strength, high crystallinity, and narrow processing window. Despite this, they have been successfully foamed by injection foaming and extrusion foaming [8,9,10,11,12], and chain extender additives (CE) have been reported to enhance foamability in biopolyesters such as polylactic acid [13,14,15] and PHAs [9,16]. On the other hand, techniques such as injection foaming, extrusion foaming, and supercritical CO_2_-assisted extrusion have been used for the production of cellular biocomposites [17,18,19,20,21], but the use of a nonwoven structure is a limiting factor for those techniques (i.e., the entanglement of fibres obtained in such a structure does not allow the required flow for processing). In this respect, a gas dissolution foaming technique can overcome these technical limitations. Gas dissolution foaming is a discontinuous (batch) foaming process, which consists of the saturation of a polymer with a physical blowing agent (gas) at a given temperature and pressure inside a high-pressure vessel, forming a single-phase polymer-gas solution. The expansion is achieved by applying a thermodynamic instability that abruptly reduces the gas solubility limit in the polymer, leading to the phase separation of the dissolved gas and promoting the nucleation and growth of cells. This thermodynamic instability can be induced by a temperature increase (temperature soak method) or by a quick pressure release (pressure quench method).

The aim of this study is to propose and explore a novel approach for the production of cellular lightweight fibre-reinforced green composites of polyhydroxybutyrate-based matrix and flax nonwoven fabric reinforcement. The goal is to achieve a regular expansion of the precursor with a homogeneously distributed porosity in the polymeric matrix, to reach a weight reduction of the composites while keeping a good fibre-matrix stress transfer and adhesion. The approach consists of foaming—by a gas dissolution process—the composite precursors in which the reinforcement is a nonwoven structure (fabric). For the foaming process, after CO_2_ dissolution at high temperature, the phase separation in the matrix is induced by a pressure quench. The viability of the system is evaluated through the analysis of the cellular structure and morphology of the composites. Preliminary tests are performed to determine the conditions in which the lowest density values are reached, where the influence of the nonwoven fabrics on the matrix’s foaming behaviour is observed. Further tests, considering variations of four factors (matrix type, nonwoven fabric’s treatment, expansion temperature, and expansion pressure) are accomplished to determine the effects of these factors on the density and cellular structure of the cellular composite samples.

## 2. Materials and Methods 

### 2.1. Foamed Composites Production

**Figure 1:**
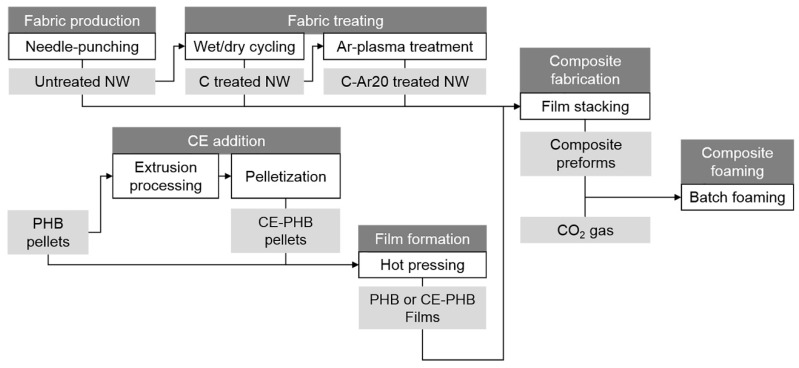
Scheme for the samples preparation.

Figure 1 summarizes the procedure followed to prepare the foamed composites. As shown, the preparation of composite precursors required firstly the production and treatment of nonwoven fabrics (NW), and secondly the production of films made of modified or non-modified polymeric matrices, further processed by film-stacking. The solid composite precursors were then foamed after the CO_2_ dissolution by a quick pressure release. A detailed description of this procedure is presented hereunder.

#### 2.1.1. Production and Treatment of the Natural Fibre Nonwoven Fabrics

The production and treatment of the flax NW fabrics (summarized in Figure 1) was addressed in a previous study [22,23]. The NW flax reinforcements with a density of 284 ± 23 g/m^2^ and a thickness of 1.9 ± 0.1 mm were used in three different conditions: as fabricated (untreated), after wet/dry cycling (C), and after wet/dry cycling and treatment with argon plasma (C-Ar20).

#### 2.1.2. CE Addition to the Matrix

The biopolymer used was Mirel P3001, a proprietary polyhydroxybutyrate (PHB)-based resin of thermoforming grade provided by Metabolix (Cambridge, MA, USA), designated hereafter as PHB. For its use in the matrix, it was used as received, as well as modified with a chain extender (CE) to improve its foamability (referred to as CE-PHB). For the production of this CE-PHB matrix, the PHB pellets were dried at 50 °C overnight, and mixed with 1 wt % of the CE Joncryl ADR-4368-C, kindly provided by BASF Española (Barcelona, Spain). The mixture was then processed in a COLLIN ZK 25 T co-rotating twin-screw extruder (Dr. Collin GmbH, Ebersberg, Germany), using a reverse temperature profile linearly decreasing from 170 °C to 150 °C at a screw-speed of 70 rpm and at a feeding rate of ~70 g/min. The extrudate was cooled in a water bath and pelletized.

#### 2.1.3. Fabrication of Solid Composite Precursors

Both PHB and CE-PHB matrices were used to produce films of 0.6 mm thickness, with the help of a frame, in a COLLIN P 300P Hot Plate Press (Dr. Collin GmbH, Ebersberg, Germany). The process consisted of heating at 175 °C for 5 min to melt the polymer and a further 5 min under a 50 MPa pressure, followed by cooling to room temperature under a 50 MPa pressure. Composites (1 mm thick) were produced in a sandwich-like sequence consisting of film/NW/film by means of the film-stacking method. The temperature was set to 175 °C, and the pressure profile consisted of 6 min under no pressure for melting the polymer, followed by 4 min under 50 MPa for the fibre impregnation, and a cooling step under 50 MPa. Six solid precursors (with an average fibre fraction of 19.8 ± 1.5 wt %) were prepared. In order to identify the composite precursors as a function of the type of matrix and reinforcement, the samples will be referred to hereafter as: untreated/PHB, untreated/CE-PHB, C/PHB, C/CE-PHB, C-Ar20/PHB and C-Ar20/CE-PHB. Five specimens of 40 mm × 60 mm were cut from each of these solid precursors.

#### 2.1.4. Foaming Process of the Solid Composite Precursors

**Figure 2:**
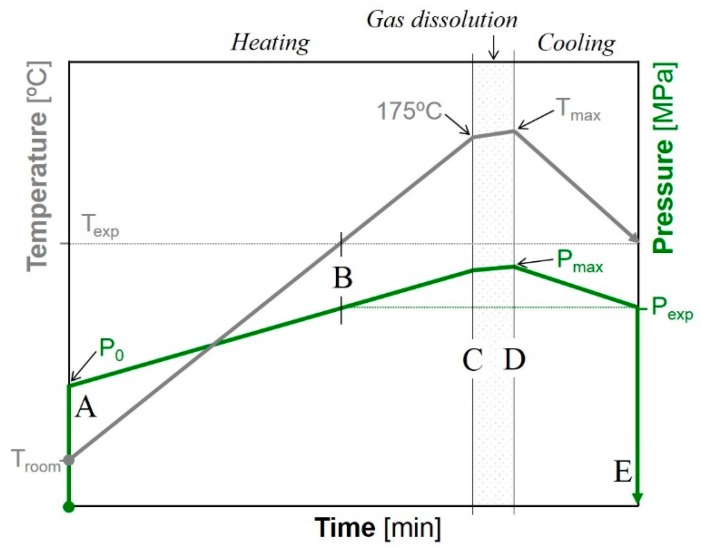
Definition of the temperature and pressure curves in the batch-foaming process.

The pressure quench foaming method was used to foam the former solid precursors, which were dried at 80 °C overnight under vacuum. The samples were placed inside the high-pressure vessel. The dissolution of the blowing agent, CO_2_ (99.9% purity) supplied by Rivoira SpA (Milan, Italy), was performed at high temperature, above the melting temperature of the polymer, to achieve the highest gas solubility available in the amorphous state. Foaming was achieved by a quick pressure release after cooling to the desired expansion temperature. For better understanding, a scheme of the pressure and temperature evolution is shown in Figure 2. 

It is worth noting that the foaming times were dependent on the heating/cooling capabilities of the experimental setup. The steps from A to D were performed as follows. (A) the pressure vessel was filled with CO_2_ at an initial pressure (*P*_o_) dependent on the required expansion pressure (*P*_exp_), where *P*_o_ was 3.75 ± 0.25 MPa, 6.25 ± 0.25 MPa, and 7.25 ± 0.25 MPa for *P*_exp_ of 5, 10, and 20 MPa, respectively. (A–C) the temperature was raised (heating), with the consequent pressure increase, up to 175 °C. The heating rates, which were affected by the CO_2_ pressure in the vessel, were determined as 5.8, 5.1, and 4.6 °C/min for expansion pressures of 5, 10, and 20 MPa, respectively. (B) During heating, the pressure was controlled and regulated, if required, to be coincident with the final targeted conditions. (C,D) After reaching 175 °C, a 3-min step was set for dissolution of the CO_2_ in the polymer. The short dissolution time is due to the sensitivity of PHAs to thermal degradation at temperatures just above their melting point. (D) Owing to the thermal inertia of the experimental apparatus, the maximum temperature (*T*_max_) and pressure (*P*_max_) were recorded at the end of the gas dissolution step. (D,E) the vessel was cooled to the targeted foaming temperature *T*_exp_ (120 or 140 °C) at a rate of −4.9 ± 0.3 °C/min (cooling). (E) Once the desired conditions were reached, the expansion was achieved by a fast pressure release at a rate of 40 MPa/s conducted by means of a gas evacuation system. The total time spent on the process was longer for the samples foamed at 120 °C than for the samples foamed at 140 °C due to the need for longer cooling from 175 °C to a lower temperature. Finally, the vessel was quickly opened and the samples extracted to avoid the modification/collapse of the cellular structure. 

### 2.2. Samples Characterisation

#### 2.2.1. Rheology Measurements

For the evaluation of the effect of the nonwoven fabric on the rheology of the composites, the dependence of viscosity on the shear rate of the two matrices (PHB and CE-PHB) and the untreated/PHB solid composite precursor was measured. The steady state flow curves at 175 °C were obtained in a TA Instruments Rheometer AR 2000 EX (TA Instruments, New Castle, DE, USA), equipped with electrically heated parallel plates of 25 mm diameter, with a gap set to 1 mm. The viscosity was measured, for comparative purposes, under shear rates between 0.001 and 10 s^−1^ in nitrogen atmosphere.

#### 2.2.2. Density

The geometric density (*ρ*) of the samples was determined by dividing the mass of the specimen by its external volume, obtained from its dimensions, before (*ρ*_solid_) and after expansion (*ρ*_foam_). The relative density (*ρ*_rel_) was calculated as *ρ*_rel_ = *ρ*_foam_/*ρ*_solid_.

#### 2.2.3. Cell Size and Cell Density

The mean cell size (*ϕ*) of the foams was determined with a user-interactive image analysis adaptation of the ASTM D3576-04 method, based on [24], from scanning electron microscopy (SEM) images of fragile fractures taken in a JEOL Scanning Electron Microscope JSM 820 (JEOL Ltd, Tokyo, Japan). 

The cell density per solid volume (*N*_0_) was determined according to Equation (1):

(1) $$N_{0}=\frac{\frac{6}{\pi \phi^{3}}V_{f}}{1-V_{f}}$$

where the porous fraction *V_f_* was calculated as *V_f_* = 1 − (*ρ*_foam_/*ρ*_solid_).

#### 2.2.4. Mechanical Properties

The flexural modulus was measured at room temperature in a three-point bending mode with a span of 20 mm by means of a Perkin-Elmer DMTA 7 equipment (Perkin-Elmer Inc., Waltham, MA, USA). The forces were adjusted to obtain an indenter displacement amplitude of 20 ± 2 μm (oscillation frequency of 1 Hz), and the static stress was 120% the dynamic stress. The values, taken 3 min after applying the loads, were an average of six measurements (three specimens measured on both sides). The specimens were of 22 mm × 6 mm, with variable thickness according to the expansion of the samples. From the tests, the specific stiffness (defined as flexural modulus divided by density, *E*/*ρ*) was determined.

## 3. Results and Discussion

### 3.1. Preliminary Tests

**Figure 3:**
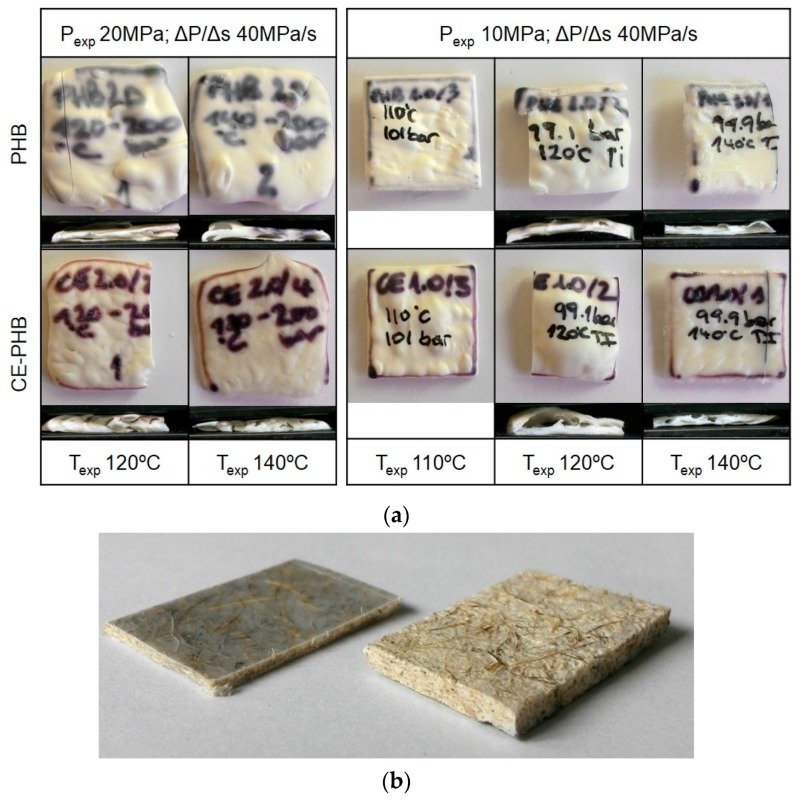
Examples of the specimens from the preliminary tests. (**a**) PHB and CE-PHB without fibres expanded and sections showing the irregularity of the cellular structure throughout the thickness; (**b**) Comparison of the untreated/PHB composites before and after expansion.

**Figure 4:**
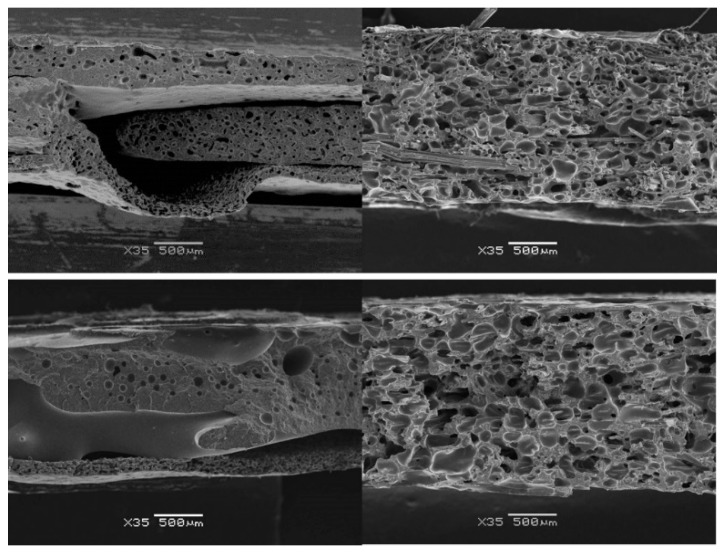
Comparison of unreinforced (**left**) and reinforced (**right**) foam sections showing large differences in the homogeneity of their cellular structure.

Preliminary tests were performed with the unreinforced polymer, with the aim of evaluating the foamability of the neat matrix in the selected experimental conditions. Some samples from this preliminary evaluation are presented in Figure 3. The expansion of unreinforced specimens, performed under a quick pressure release at 40 MPa/s, was unsuccessful. The samples were irregular and presented uneven surfaces, with some large bubbles or huge air-traps and a cellular structure that was highly heterogeneous throughout the sample thickness, as shown in Figure 3a and Figure 4 (left images). On the contrary, the composites reached very homogeneous expansions regardless of the conditions used (Figure 3b). For preliminary tests on composites, *T*_exp_ ranging from 100 °C to 170 °C, and *P*_exp_ of 5, 7.5, 10, and 20 MPa were tested. 

**Figure 5:**
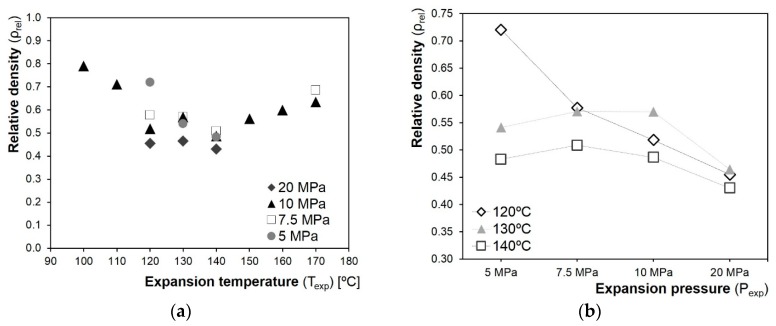
Relative density values of the untreated/PHB composite samples foamed on the exploratory tests against *T*_exp_ (**a**) and *P*_exp_ (**b**). In (**b**), the purpose of the lines is to guide the eye, and the *P*_exp_ axis is categorical (not in scale).

The exploratory results are presented in Figure 5. In Figure 5a, the relative densities achieved are plotted against the expansion temperature. The effect of *T*_exp_ on the relative density was investigated at a fixed expansion pressure of 10 MPa to restrict the temperatures at which to perform the foaming experiments. The relative density decreased with the increase of the temperature, reaching the minimum values between 120 and 140 °C, and then it started to rise again. Such temperatures were selected for the foaming experiments. In Figure 5b, the relative density results for the tests performed at 120, 130, and 140 °C at different *P*_exp_ are presented. The lowest densities were achieved at the highest expansion pressure of 20 MPa, regardless of the *T*_exp_ used. Densities ranging between 0.45 and 0.6 were obtained under 10 or 7.5 MPa at each foaming temperature, while under 5 MPa a high relative density was shown at *T*_exp_ = 120 °C. No clear differences could be observed between 7.5 and 10 MPa, and hence the 7.5 MPa expansion pressure was not further considered for the following tests.

The previous results were used to design the following experiments. The parameters considered for evaluating the foamability of the composites were the polymer matrix (PHB and CE-PHB), the nonwoven fabric type (untreated, C, and C-Ar20), and the expansion conditions. According to the foamability range observed in Figure 5, two temperatures and three pressures were considered to define the foaming conditions of the cellular composites. In particular, expansions were made under 10 and 20 MPa at 120 °C, and under 5, 10, and 20 MPa at 140 °C. All those parameters combined gave rise to 30 different experimental conditions. The effects of all these factors on the properties of the cellular composites are discussed in Section 3.3.

### 3.2. Influence of Fibres on the Foaming Process

Neat matrices revealed some difficulties in foaming under the experimental conditions used. As aforementioned, very irregular shapes with large voids and inhomogeneous cellular structures were observed. The low quality of the cellular morphology in the matrices was mainly attributed to the low viscosity, owing to the high temperatures required for the foaming process [25] and the *T*_m_ reduction due to the CO_2_ sorption [26], and to the reduced capability of stabilizing the developed cellular structure. In particular, the developed cellular structure, fostered by the phase separation of the gas after the quick pressure release, could not be uniformly stabilized after cell nucleation and growth by the increase of the polymer viscosity alone, and cell coalescence, collapse, or both, took place. 

Nevertheless, under the same conditions, very good foaming of the composites was easily achieved, showing a uniform expansion, homogeneous cellular morphology, and relative densities ranging between 0.45 and 0.6 for most of the specimens expanded at *T*_exp_ equal to 120 and 140 °C and *P*_exp_ range between 5 and 20 MPa. The comparison of the cellular structures of some unreinforced foams and their cellular composite counterparts (examples in Figure 4) showed smaller cells (on average) in the unreinforced foams, but these did not develop a regular structure. Large voids were present and a largely irregular morphology was detected. The lower apparent mean cell size can be related to the migration of gas into the large voids and, consequently, most of the cells collapsed and reduced their cell size. On the contrary, the fibres had a clear effect on the cell growth, playing a key role in the stabilization of the morphology and in hindering cell collapse. It must be pointed out that the homogeneous distribution of fibres in the precursor was a key parameter, since areas with low fibre content experienced the same issues as neat matrices. According to these results, the improved foamability in the presence of fibres can be attributed to an increase of the overall viscosity, due to the polymer flow being hindered, and to a stabilizing effect of the fibre network after cell growth. 

**Figure 6:**
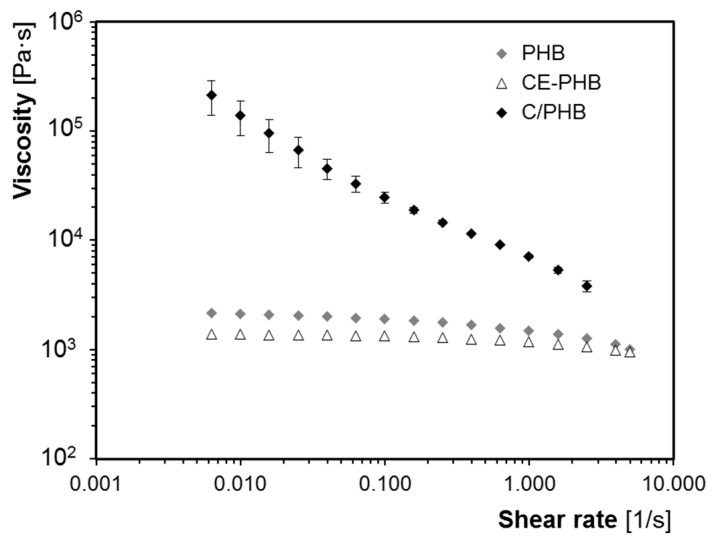
Steady state flow curves for the solid materials with and without nonwoven fabric reinforcement.

A rheological characterisation was performed in the PHB, CE-PHB and C-PHB (precursor) samples in order to determine possible changes in the viscosity. The steady state flow curves are shown in Figure 6. 

As can be observed, the viscosity curve for the fibre-reinforced material is around two orders of magnitude higher compared to the unreinforced matrices. Therefore, the addition of fibres clearly limited the polymer flow of the material in the melt state, and the contribution of the viscosity increase led to a higher foamability in terms of the regularity of the cellular morphology throughout the thickness for the composite samples. 

As aforementioned, all cellular composite samples showed similar relative densities. By doubling the expansion pressure from 10 MPa to 20 MPa, the density values were barely lowered. This could point to a capping effect of the flax fabric reinforcement, which could be helping to achieve a regular foaming although limiting the maximum expansion at the same time. This degree of constriction could be a consequence of the mechanical entanglements of long fibres in the NW structure, produced by its own fabrication method where a 3D fibre network is obtained. Moreover, since flax fibres are stable at the *T*_m_ of the matrix (~174 °C) and cannot flow, the structural integrity of NWs remains throughout almost all of the batch-process, this guaranteeing the shape stability of the specimens. 

### 3.3. Influence of the Processing Conditions on the Cellular Composites’ Properties

#### 3.3.1. Density

**Figure 7:**
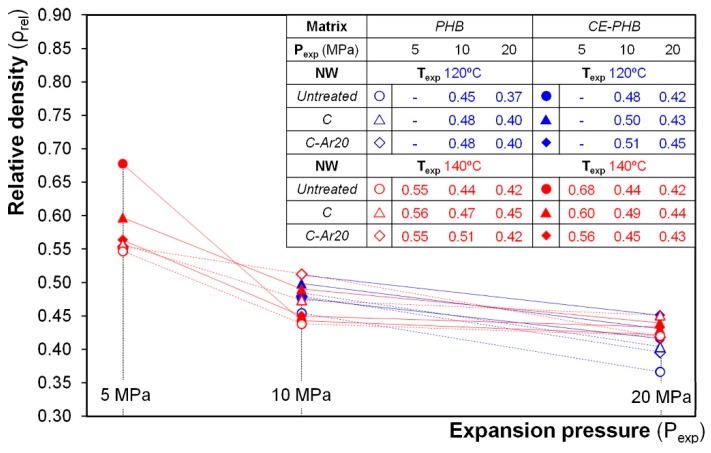
Relative densities of the cellular composites obtained according to the variables of study. The lines only serve to guide the eye.

The results of relative density achieved against the expansion pressure are plotted in Figure 7. Specimens foamed at *P*_exp_ equal to 10 and 20 MPa presented relative densities ranging from 0.45 to 0.5 and from 0.4 to 0.45, respectively. 

Significantly higher densities (i.e., a lower expansion) and variability were obtained at *P*_exp_ = 5 MPa. This can be related to the lower solubilisation pressure during the gas dissolution process. The lower amount of blowing agent available has multiple effects: (a) reduces the maximum expansion ratio achievable; (b) reduces the thermodynamic instability during the pressure quench (thus reducing the nucleation of cells); (c) results in a weaker viscosity reduction (due to the lower amount of plasticisation) and hence in higher forces to be overcome by the growing bubble. The higher amount of absorbed CO_2_ in the polymers at *P*_exp_ = 10 MPa lowered the density, thus levelling the expansion ratio, and an even higher solubilisation pressure of 20 MPa (albeit lowering the viscoelastic properties of the polymer) did not result in a larger density reduction. This limited density reduction under *P*_exp_ = 20 MPa with respect to *P*_exp_ = 10 MPa could be attributed to the effect of the reinforcement structure, owing to the degree of constriction explained before.

#### 3.3.2. Cellular Structure

**Figure 8:**
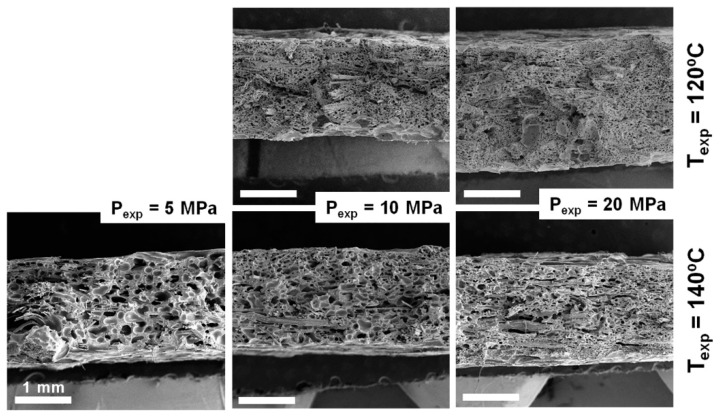
SEM images of the C-Ar20/PHB composite series. Scale bars correspond to 1 mm.

To evaluate the cellular structure, images of fractures (observed by SEM) of the 30 specimens were analysed. Some examples are presented in Figure 8, where the cellular structure throughout the thickness can be observed.

In general terms, a good distribution of the fibres through all the specimens was observed. This homogeneity was attributed to the NW nature of the reinforcement fabric and a good impregnation achieved in the precursors during the film-stacking. On the other hand, a good overall adhesion was observed (and hence a good load transfer is expected), although all the specimens presented some fibre pull-out, which pointed to certain heterogeneities in the fibre-matrix adhesion. 

**Figure 9:**
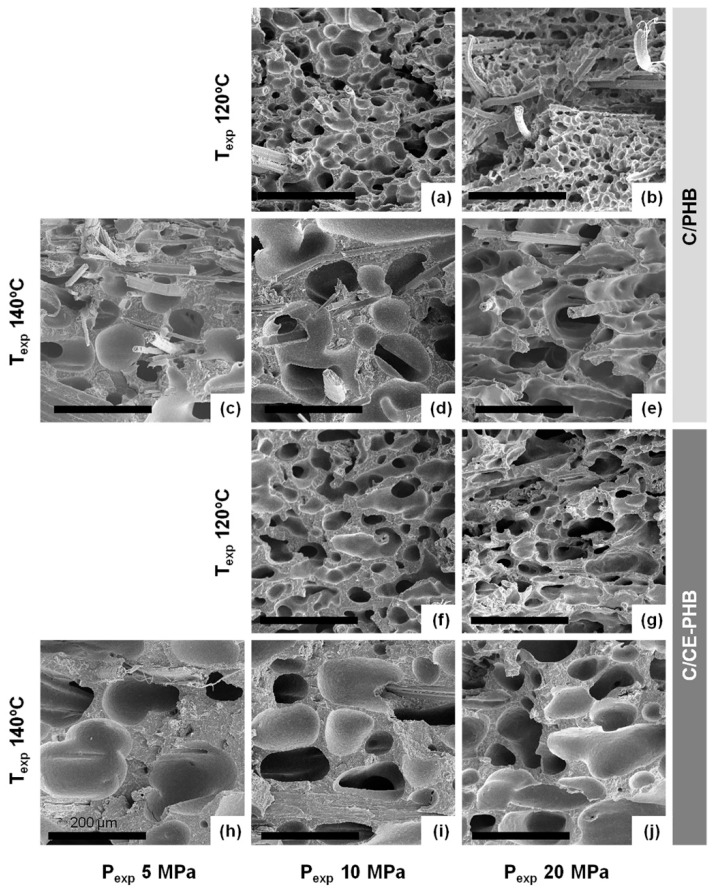
SEM images of the (**a**–**e**) C/PHB series and (**f**–**j**) C/CE-PHB series, as an example of the different cellular structures observed regarding the different expansion conditions. Scale bars correspond to 200 μm.

The morphology of the cellular structures at a higher magnification is presented in Figure 9, in order to show the clear differences observed in the cell size and the cell density. In general, the increase of the expansion pressure led to a reduction of cell size and a higher cell density for all the systems. The higher amount of gas dissolved was translated not only into a higher cell nucleation, but also into an increasing cell coalescence, given the low capability of the polymer to withstand extensional stresses and the higher available gas after the higher solubilisation pressure. The overall effect on cell size and cell density of such competing parameters seemed to be primarily governed by temperature and pressure, and to a lesser extent by cell coalescence.

**Figure 10:**
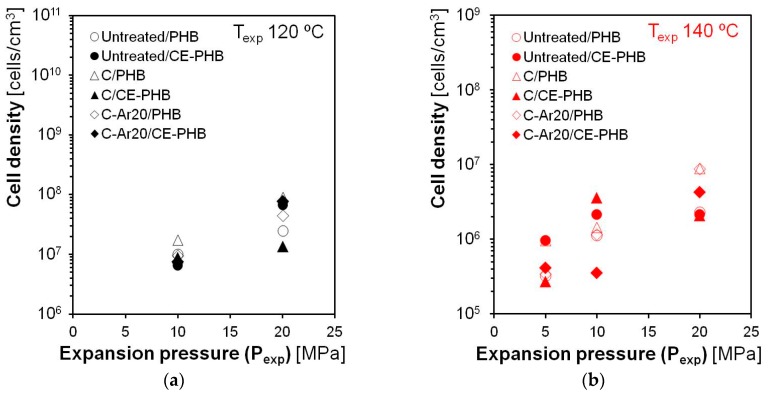
Cell densities of the cellular composites obtained according to the variables of study for the composites expanded at 120 °C (**a**) and at 140 °C (**b**).

Results on the cell density for *T*_exp_ = 120 °C and *T*_exp_ = 140 °C have been plotted against *P*_exp_ in Figure 10, showing a clear influence of both the temperature and pressure on the amount of nucleated cells. The cell density decreased with the increase of temperature (in the range of 10^5^–10^7^ cells/cm^3^ at 140 °C and 10^6^–10^8^ cells/cm^3^ for 120 °C) but increased with the increase of *P*_exp_, reaching maximum values for the samples expanded at 20 MPa. This increase of nucleated cells with the solubilisation pressure (related to the expansion pressure) is in accordance with the classical theory of bubble nucleation, which predicts a larger amount of bubbles with the increase of the blowing agent content at constant temperature and the thermodynamic instability (pressure decrease to induce foaming). On the other hand, the increase of temperature at the same time lowered the viscosity, which favours the cell growth but also the coalescence of bubbles due to the low capability of the investigated polymer to bear extensional stresses, leading to a reduction of cell density.

While the cellular structure was highly influenced by the processing conditions for foaming, the fibre treatments and the presence of CE did not seem to affect the cellular structure in the processing conditions used in the present work. The modifications applied to the fibres did not foster the formation of the cellular structure. On the other hand, as shown in Figure 6, the CE addition led to a lower viscosity of the CE-PHB matrix (possibly due to the degradation derived from the extra thermal processing required), which would explain the lack of improvement in the cellular morphology.

#### 3.3.3. Mechanical Behaviour

As can be observed in Figure 8 and Figure 9, the specimens presented a good distribution of the fibres, which were well embedded in the matrix (mainly inside the cell walls). The good adhesion promotes high load transfer capability. In Table 1, the specific stiffness values are presented. 

The addition of CE increased the stiffness of the matrix (the specific stiffness was ~30% higher). For that reason, the specific stiffness of all the CE-PHB-based composite precursors was higher than for those with PHB matrix. Additionally, the presence of fibres increased the specific stiffness of the composite precursors by around 60% with respect to the unreinforced matrices. The reinforcement type revealed some differences in the specific stiffness of the solid precursors, showing the best overall results for those with the C treatment. 

Regarding the cellular composites, the specific stiffness for the foams was equal to or higher than for the unreinforced matrix, although lower than the specific stiffness of the solid precursors, due to the effect of the cellular structure (the properties of cellular materials decrease with density). The C/PHB and C-Ar20/CE-PHB cellular composites presented the best overall results among foams.

The properties of the cellular materials obtained and the good regularity of the foaming method proposed point to a possible industrial use, with potential applications in the automotive industry (e.g., for interior door panels), biodegradable trays for packaging or as sustainable alternatives for lightweight structural panels, for instance.

## 4. Conclusions

The gas dissolution/pressure quench foaming of PHB-based matrix with flax nonwoven fabric reinforcement showed a high regularity in the density reduction, with homogeneous cellular morphology and a uniform distribution of the fibres, which were found to be embedded in the cellular matrix. Furthermore, an overall good fibre–matrix adhesion was achieved, all thus resulting in a good stress transfer as evidenced by the high specific stiffness measured.

The presence of the fibres increased the viscosity of the composites in the melt state. The presence of the nonwoven structure played a key role in stabilizing the cellular morphology regardless of the treatment applied. Moreover, the use of the chain extender increased the stiffness of the matrix, leading to higher specific stiffness of both solid precursors and cellular composites.

The foaming parameters (foaming temperature and expansion pressure) influenced the developed cellular structure and the density reduction. The cell size increased with the temperature but decreased with the expansion pressure. Relative densities around 0.4–0.5 were achieved, with the lowest values obtained when expanding the samples at 140 °C and 20 MPa. It is worth noting that the use of a higher pressure had a marginal effect with respect to 10 MPa.

The cellular composites produced presented a porosity of ~50% and a fibre content of ~20 wt %, both of which can contribute to the reduction of the cost of PHA-matrix-based materials, and offered higher specific properties with respect to the neat polymer, thus enhancing its competitiveness.

## Figures and Tables

**Table 1:** Flexural modulus and relative density of some samples.

Reference	Sample Type	*T*_exp_ (°C)	*P*_exp_ (Mpa)	Specific Stiffness, *E*/*ρ* (GPa/g·cm^−3^)
PHB	Matrix	-	-	1.11
Untreated/PHB	Precursor	-	-	1.78
	Foam	120	10	1.40
		140	10	1.47
		140	20	1.43
C/PHB	Precursor	-	-	1.75
	Foam	120	10	1.81
		140	10	1.74
		140	20	1.44
C-Ar20/PHB	Precursor	-	-	1.66
	Foam	120	10	1.23
		140	10	1.50
		140	20	1.43
CE-PHB	Matrix	-	-	1.41
Untreated/CE-PHB	Precursor	-	-	2.21
	Foam	120	10	1.48
		140	10	1.58
		140	20	1.37
C/CE-PHB	Precursor	-	-	2.42
	Foam	120	10	1.62
		140	10	1.64
		140	20	1.45
C-Ar20/CE-PHB	Precursor	-	-	2.04
	Foam	120	10	1.70
		140	10	1.65
		140	20	1.65
